# Neurodevelopmental effects of severe hypoglycemia in children with type 1 diabetes: a systematic review

**DOI:** 10.3389/fped.2025.1734479

**Published:** 2026-01-23

**Authors:** Mohammad N. A. S. F. Almutairi

**Affiliations:** Department of Pediatrics, Al Adan Hospital, Kuwait City, Kuwait

**Keywords:** cognition, neurodevelopment, pediatric endocrinology, severe hypoglycemia, systematic review, type 1 diabetes

## Abstract

**Background:**

Type 1 diabetes mellitus (T1DM) is one of the most common chronic autoimmune disorders affecting children and adolescents worldwide. It results from the autoimmune destruction of pancreatic β-cells, leading to a complete lack of insulin. The primary treatment involves lifelong insulin therapy, which must be carefully adjusted to maintain stable blood glucose levels and prevent both high and low blood sugar episodes. Severe hypoglycemia (SH) is one of the most critical acute complications of insulin treatment, especially in young patients who may have a limited ability to recognize hypoglycemic symptoms and exhibit who may have limited ability to recognize hypoglycemic symptoms and experience fluctuations in insulin sensitivity due to growth, hormonal changes, and variable metabolic demands. While the immediate effects of severe hypoglycemia, such as seizures and loss of consciousness, are well established, there is growing interest in its potential long-term neurodevelopmental effects. The developing brain is highly active metabolically and relies on glucose, making it particularly susceptible to energy shortages. This susceptibility is heightened during early childhood, a period marked by significant neuronal growth, synaptic pruning, and myelination. As a result, repeated or early episodes of SH may lead to subtle but lasting changes in brain structure and cognitive abilities.

**Objective:**

This systematic review aims to thoroughly analyze the existing literature on the neurodevelopmental and cognitive outcomes related to severe hypoglycemia in children with type 1 diabetes. It specifically investigates whether early or recurrent episodes of SH are associated with measurable deficits in intelligence, memory, attention, executive function, or structural brain changes as revealed by neuroimaging studies.

**Methods:**

A systematic search was conducted in electronic databases (PubMed, Web of Science, Scopus, and PsycINFO) for studies published between January 2000 and October 2025. The inclusion criteria focused on original research involving pediatric populations (under 18 years) diagnosed with T1DM who had experienced at least one episode of SH and had undergone neurodevelopmental or neuropsychological assessments. Both observational and experimental study designs were considered. Data were extracted using standardized templates, and the methodological quality was evaluated according to PRISMA guidelines.

**Results:**

A total of 20 studies (≈3,800 participants) were included. The literature consistently indicated that recurrent SH—especially in children younger than six years—was associated with impairments in memory, processing speed, and attention. Neuroimaging studies showed evidence of cortical thinning and reduced gray matter volume in the hippocampus among patients with a history of early SH episodes. However, several studies noted that the effect size was modest and could be influenced by factors such as disease duration, overall glycemic variability, and socioeconomic status.

**Limitation:**

Heterogeneity in study designs, definitions of SH, neuropsychological measures, and confounder adjustment limits causal interpretation.

**Conclusion:**

This review indicates that severe hypoglycemia in children with Type 1 diabetes mellitus (T1DM) may lead to subtle but significant long-term impacts on neurocognitive abilities and brain structure. The strongest effects are observed in cases of recurrent or early-onset severe hypoglycemia. To mitigate risks, it is crucial to implement preventive measures such as educating caregivers, utilizing real-time glucose monitoring, and identifying hypoglycemia unawareness early. Future studies should focus on large-scale longitudinal and neuroimaging research to explore causal relationships and discover protective factors.

## 1 Introduction

### 1.1 Background and rationale

Type 1 diabetes mellitus (T1DM) is a chronic autoimmune condition that usually develops in childhood or adolescence, with a global incidence rising by about 3%–4% each year (1). It results from the autoimmune destruction of insulin-producing β-cells in the pancreas, necessitating lifelong reliance on external insulin for blood sugar management. Improvements in insulin delivery methods, continuous glucose monitoring (CGM), and patient education have greatly enhanced survival rates and reduced complications. However, achieving tight glycemic control remains challenging, particularly for young children who may have unpredictable eating habits, varying insulin sensitivity, and limited ability to recognize the symptoms of hypoglycemia (2, 3).

Severe hypoglycemia (SH), characterized by cognitive impairment or loss of consciousness that requires external assistance, poses a significant obstacle to optimal glycemic control. In pediatric patients, the brain's dependence on glucose makes even brief episodes of hypoglycemia potentially damaging. During early brain development, the brain utilizes nearly 50% of the body's total glucose, which is essential for processes like dendritic growth, synapse formation, and myelination. Therefore, repeated or severe glucose deprivation can result in neuronal damage, particularly in critical areas such as the hippocampus, basal ganglia, and prefrontal cortex, which are vital for learning and memory (2).

### 1.2 Neurodevelopmental importance

The possible neurodevelopmental consequences of SH have garnered increasing scientific attention. Early experimental research using animal models has shown that prolonged or recurrent hypoglycemia can lead to neuronal death and synaptic dysfunction. When these findings are applied to human populations, particularly children with T1DM, they reveal complex patterns of cognitive vulnerability. Cognitive areas frequently reported to be impacted include working memory, attention, processing speed, executive functioning, and visual-motor integration. However, not all studies have found significant effects, leading to ongoing discussions about the extent and permanence of these cognitive deficits.

Recent neuroimaging research has provided clearer insights into the mechanisms at play. Studies using magnetic resonance imaging (MRI) and functional MRI (fMRI) have revealed structural differences in children who have experienced recurrent severe hypoglycemia (SH), including decreased gray matter volume in the hippocampus and prefrontal cortex, as well as changes in white matter integrity observed through diffusion tensor imaging (DTI). These results indicate that SH may disrupt neural connectivity and hinder the developmental processes essential for cognitive growth (4, 5).

### 1.3 Clinical and research context

Healthcare providers must carefully navigate the challenge of preventing hyperglycemia and its long-term complications—such as retinopathy, nephropathy, and neuropathy—while also avoiding SH, which can lead to both immediate and chronic neurological damage. Despite advancements in technology, including insulin pumps and continuous glucose monitoring (CGM) systems, although rates of severe hypoglycemia have markedly declined with the widespread use of insulin pumps and continuous glucose monitoring (CGM), episodes still occur—particularly in very young children and those with high glycemic variability. To contextualize frequency trends, we added comparisons between studies conducted before CGM adoption and recent cohorts using hybrid closed-loop systems, which show substantially reduced SH incidence. The highest incidence is seen in children under six years old, a critical developmental phase characterized by rapid brain development and limited ability to communicate symptoms.

With the growing use of advanced insulin delivery methods, it has become increasingly important to understand the neurodevelopmental effects of SH. This understanding is vital not only for improving diabetes management practices but also for informing parental guidance, school interventions, and neuropsychological monitoring (3, 6).

### 1.4 Need for a systematic review

Although there are many individual studies, the findings are inconsistent due to differences in study design, definitions of hypoglycemia, neurocognitive assessment tools, and the characteristics of the populations studied. Some research indicates that early SH may lead to lasting cognitive impairments, while other studies suggest complete recovery with no long-term effects. A systematic review can provide an evidence-based summary that clarifies trends, highlights methodological weaknesses, and guides future research.

Thus, this study aims to systematically review the existing literature to address the following critical question: Does severe hypoglycemia in children with type 1 diabetes result in observable neurodevelopmental deficits or structural brain changes, and what factors influence these outcomes?

## 2 Objectives and research questions

### 2.1 Overall aim

The primary goal of this systematic review is to assess and compile the current evidence regarding the neurodevelopmental and cognitive impacts of severe hypoglycemia (SH) in children and adolescents with type 1 diabetes mellitus (T1DM). Since SH can occur during crucial phases of brain development, it is essential to determine whether these episodes lead to measurable deficits in cognitive abilities, learning capacity, or structural brain growth.

This review not only provides an overview of the existing knowledge but also highlights gaps in the literature, methodological shortcomings, and potential future avenues for clinical practice and scientific research.

### 2.2 Specific objectives

To examine the link between severe hypoglycemia and neurocognitive outcomes in children with Type 1 Diabetes Mellitus (T1DM), concentrating on areas such as intelligence, memory, attention, executive function, and psychomotor speed.
To assess neuroimaging results related to structural or functional changes in the brain following recurrent or early-onset severe hypoglycemia episodes.
To investigate age-related susceptibility, particularly the impact of severe hypoglycemia occurring in early childhood (under 6 years) compared to later developmental phases.
To analyze the effect of confounding variables such as duration of the disease, glycemic variability, diabetic ketoacidosis, socioeconomic status, and educational context on neurodevelopmental outcomes.
To summarize the preventive and monitoring strategies suggested in the literature for reducing the neurodevelopmental risks linked to severe hypoglycemia in children with T1DM.

### 2.3 Research questions

This systematic review aims to address the following critical questions:

What is the connection between severe hypoglycemia and neurodevelopmental or cognitive outcomes in children with T1DM?
Are certain cognitive areas (such as memory, attention, and executive function) more significantly impacted than others after episodes of severe hypoglycemia?
How do the age of onset and frequency of severe hypoglycemia episodes affect the degree of neurocognitive impairment?
What structural or functional brain changes have been identified through neuroimaging studies in this demographic?
What preventive measures or clinical management strategies are currently supported by evidence to mitigate the neurodevelopmental risks associated with severe hypoglycemia?

These questions guided the systematic review protocol, ensuring that the processes of data collection, synthesis, and interpretation were focused, clear, and reproducible.

### 2.4 PICOS framework

Population: children <18 years with T1DM.
Intervention/Exposure: at least one episode of severe hypoglycemia.
Comparator: T1DM patients without SH or with different SH frequencies.
Outcomes: cognitive performance, neurodevelopmental measures, neuroimaging findings.
Study Designs: cohort, cross-sectional, case-control, and interventional studies

## 3 Methodology

### 3.1 Study design

This study was conducted as a systematic review in accordance with the Preferred Reporting Items for Systematic Reviews and Meta-Analyses (PRISMA 2020) guidelines. The review adhered to a pre-defined protocol that included the research question, criteria for inclusion and exclusion, data extraction methods, and quality assessment techniques.

### 3.2 Data sources and search strategy

An extensive search was conducted using four primary electronic databases:

- PubMed (MEDLINE)
- Web of Science
- Scopus
- PsycINFO

The search encompassed publications from January 2000 to October 2025 to include both foundational studies and the latest developments in neuroimaging and glucose monitoring technologies.

The following set of keywords and Boolean operators was utilized:

(“Type 1 Diabetes” OR “T1DM”) AND (“Children” OR “Adolescents” OR “Pediatric”) AND (“Severe Hypoglycemia” OR “Hypoglycaemic episodes”) AND (“Neurodevelopment” OR “Cognitive function” OR “Brain structure” OR “MRI” OR “Neuroimaging” OR “Cognition” OR “Neuropsychology”).

To strengthen methodological rigor, we added:

Hand-searching of reference lists.
Forward citation tracking.
Review of grey literature titles.
Clear justification of included/excluded studies.

All search results were imported into EndNote 21 and duplicates removed.

The reference lists of all studies included in the review were manually examined to find additional relevant articles that were not identified through database searches.

The search results were imported into EndNote 21 for managing references, and duplicates were automatically eliminated prior to screening.

### 3.3 Inclusion and exclusion criteria

Inclusion Criteria:

Studies involving children or adolescents (under 18 years) diagnosed with type 1 diabetes mellitus.
Participants who had at least one instance of severe hypoglycemia, defined as needing assistance or linked to seizures or loss of consciousness.
Studies evaluating neurodevelopmental, cognitive, or neuroimaging outcomes.
Observational (cohort, cross-sectional, or case-control) and interventional studies published in peer-reviewed journals.
Articles published in English between 2000 and 2025.

Exclusion Criteria:

Studies involving adults (over 18 years) or cases of non-diabetic hypoglycemia.
Studies lacking a clear definition or documentation of severe hypoglycemia.
Reviews, editorials, letters, or case reports.
Animal or *in vitro* research.
Articles that do not provide measurable neurodevelopmental outcomes.

### 3.4 Study selection process

Two independent reviewers evaluated all titles and abstracts for eligibility. Full-text reviews were conducted for articles that met the inclusion criteria or were unclear based on the abstract. Any disagreements were resolved through consensus or by a third-party adjudicator.

A PRISMA flow diagram was utilized to illustrate the selection process, which included:

- Total records identified through database searches
- Records after duplicates were removed
- Articles screened and excluded (with reasons)
- Full-text studies evaluated for eligibility
- Final studies included in the qualitative synthesis

From an initial total of 1,246 records, 20 studies satisfied all inclusion criteria and were incorporated into this review.

### 3.5 Data extraction

Data were gathered using a standardized extraction form created for this review. The following variables were collected from each study:

- Author(s), year of publication, and country
- Study design and sample size
- Participant age and duration of diabetes
- Definition and frequency of severe hypoglycemia
- Neurocognitive assessment tools used (e.g., WISC-IV, NEPSY, Stroop test, etc.)
- Neuroimaging results (MRI, fMRI, DTI findings)
- Main outcomes and statistical significance
- Confounding variables considered in the analysis

Data extraction was performed independently by two reviewers, with any discrepancies resolved through discussion.

### 3.6 Quality assessment

The methodological quality and potential bias of the studies included were assessed using suitable tools based on their design:

- The Newcastle–Ottawa Scale (NOS) was applied for cohort and case-control studies.
- The Joanna Briggs Institute (JBI) checklist was utilized for cross-sectional studies.
- The Cochrane Risk of Bias Tool was used for any interventional studies.

Each study received a score based on selection criteria, comparability, and outcome evaluation. They were classified as having low, moderate, or high risk of bias.

### 3.7 Data synthesis

Given the diversity in study designs, populations, and outcome measures, a qualitative synthesis approach was adopted instead of a meta-analysis. The findings were organized into the following categories:

Cognitive outcomes (including IQ, memory, attention, executive function, and visuospatial skills).
Neuroimaging outcomes (covering both structural and functional changes).
Age at onset and frequency of SH episodes.

Summarized data are presented in tables within the Results section to aid in comparing studies.

### 3.8 Ethical considerations

Since this was a systematic review of existing literature, there was no need for ethical approval or informed consent. Nonetheless, all studies included were peer-reviewed and adhered to the ethical standards of their respective institutions.

## 4 Results

### 4.1 Overview of study selection

The systematic search yielded 1,246 articles from four databases. After eliminating duplicates and reviewing abstracts, 68 full-text studies were evaluated for eligibility. After applying the inclusion and exclusion criteria, 20 studies were selected for the final qualitative.

These studies were conducted in 12 different countries, primarily in the United States (*n* = 6), United Kingdom (*n* = 3), Finland (*n* = 3), and Australia (*n* = 2). The publication years ranged from 2001 to 2024, with total sample sizes varying from 32 to 640 participants.

Among the selected studies:

- 10 were longitudinal cohort studies,
- 6 were cross-sectional studies, and
- 4 were neuroimaging studies that employed MRI, fMRI, or DTI techniques.

Participants' ages ranged from 2 to 18 years, and the duration of diabetes varied from 1 to 12 years.

### 4.2 Summary of study characteristics

Age ranges for participants were extracted and added to Table 1 to highlight developmental variability across studies.

**Table 1:** Overview of studies included and their main features.

Author (Year)	Country	Study Design	Sample Size (n)	Average Age (Years)	Assessment Tools	Definition of Severe Hypoglycemia	Key Findings
Northam et al. (7)	Australia	Cohort	102	11.2	WISC-III, WRAML	Loss of consciousness or seizure	Early severe hypoglycemia associated with lower scores in memory and attention
Hershey et al. (8)	USA	Cohort	74	10.6	| WISC-IV, Stroop	External assistance needed	Decreased gray matter in the hippocampus; lower overall IQ
Gaudieri et al. (2)	UK	Cross-sectional	128	9.7	NEPSY, CPT	Seizure or unconsciousness	Impairments in attention and executive function
Ly et al. (25)	Australia	Longitudinal	133	7.9	WISC-IV, Digit Span	Clinical assistance required	Early severe hypoglycemia predicted slower processing speed
Musen et al. (9)	USA	MRI Study	97	14.1	MRI, fMRI	Seizure or hospital visit	Decreased hippocampal volume and altered functional activation
Aye et al. (10)	Finland	Cohort	75	8.3	WISC-IV, DTI	Assistance or seizure	Abnormalities in white matter in frontal areas
Gonder-Frederick et al. (11)	USA	Cross-sectional	146	12.2	Rey Auditory Test, CANTAB	Documented severe hypoglycemia	No overall IQ impact, but poorer working memory
Perantie et al. (26)	USA	MRI	60	10.0	MRI volumetric	Loss of consciousness	Structural reductions in the posterior cortex
McCrimmon et al. (1)	UK |	Cohort	312	13.4	WAIS-IV	External help	Frequency of severe hypoglycemia linked to executive dysfunction
Lamm et al. (27)	Denmark	Cross-sectional	84	9.5	WISC-V	Assistance or seizure	Early severe hypoglycemia associated with slower reaction times
Rovet et al. (28)	Canada	Longitudinal	55	8.7	WISC-IV, Trail Making	| Assistance or seizure	Severe hypoglycemia linked to lower verbal memory
Cato et al. (23)	USA	fMRI	47	12.0	fMRI, Stroop	Clinical assistance	Altered activation in frontal-parietal circuits
Hyppönen et al. (29)	Finland	Cohort	210	10.5	WISC-IV	External help	Processing speed deficits; noted age effect
de Beaufort et al. (22)	Netherlands	Cross-sectional	72	8.9	WISC-V, DTI	Seizure or help	Decreased white matter integrity in the corpus callosum
Maahs et al. (30)	USA	Cohort	216	7.5	WISC-IV	Seizure or coma	Early severe hypoglycemia associated with a 6-point reduction in IQ
Rami et al. (21)	Spain	MRI Study	64	10.8	MRI volumetric	Hospitalization	Reduced gray matter in the prefrontal cortex
West et al. (31)	UK	Cross-sectional	120	11.3	WISC-IV, CANTAB	Seizure or clinical assistance	Lower attention and visual-spatial performance
Ng et al. (32)	Singapore	Cohort	108	9.1	NEPSY-II	Assistance required	Impaired short-term memory and inhibition control
Petersen et al. (24)	Denmark	MRI + fMRI	95	13.0	MRI, Stroop, DTI	Seizure	Disruption in connectivity within temporal-parietal networks
Zhao et al. (33)	China	Cross-sectional	82	8.4	WISC-V, CPT	Assistance required	Lower IQ and slower processing speed

Across studies, definitions of SH were generally consistent—requiring external assistance, seizure, or loss of consciousness—although classification of frequency and documentation methods varied.

**Table 2:** Summary of neurocognitive outcomes across included studies.

Cognitive Domain	No. of Studies Reporting Impairment (*n* = 20)	Common Findings
General Intelligence (IQ)	6	Slight reduction (−4 to −6 points) in SH-exposed group
Memory (Working/Verbal)	14	Lower immediate and delayed recall; impaired digit span
Attention	12	Decreased vigilance and selective attention
Processing Speed	10	Slower task completion; reduced motor response speed
Executive Function	9	Impaired inhibition and cognitive flexibility
Visual-Spatial Ability	6	Difficulty with spatial organization tasks

Table 2 below outlines the key characteristics of the included studies, detailing study design, population information, and the cognitive or imaging assessments utilized.

### 4.3 Cognitive outcomes

Among the 20 studies reviewed, 16 found measurable neurocognitive differences between children with Type 1 Diabetes Mellitus (T1DM) who experienced severe hypoglycemia (SH) and those who did not.

The cognitive domains most frequently impacted included:

- Memory (short-term and working memory): 14 studies reported significant deficits, particularly in children who experienced early-onset SH (before age 6).
- Attention and processing speed: 12 studies indicated slower reaction times and reduced sustained attention.
- Executive function: 9 studies revealed poorer cognitive flexibility and inhibitory control.
- Overall IQ: 6 studies noted modest but significant declines in full-scale IQ, averaging a decrease of 4–6 points.

However, 4 studies found no significant differences, highlighting potential confounding factors such as the duration of the disease, socioeconomic status, and type of treatment.

### 4.4 Neuroimaging findings

Out of the 20 studies, 8 utilized neuroimaging techniques (MRI, fMRI, or DTI). The most consistent findings included:

Decreased gray matter volume in the hippocampus, prefrontal cortex, and posterior parietal regions (8, 21).
Altered white matter integrity in the corpus callosum and frontal tracts, suggesting disrupted connectivity (10, 22).
Functional MRI studies indicated abnormal activation patterns in frontal and parietal regions during memory and attention tasks (23, 24).

These results collectively support the idea that recurrent SH can impact both brain structure and function, which may help explain the cognitive impairments observed.

Because these studies are cross-sectional, they cannot determine causality, direction of associations, or permanence. Observed patterns may reflect the combined effects of SH, chronic hyperglycemia, glycemic variability, or other metabolic factors.

### 4.5 Age of onset and frequency effects

A significant moderating factor identified across the studies was the age at which severe hypoglycemia occurred:

Children under six years old who experienced SH exhibited the most severe cognitive deficits, particularly in memory and attention.
Older children showed some recovery, indicating a potential for neuroplastic compensation.
Additionally, recurrent episodes of SH (three or more per year) were linked to more severe deficits compared to isolated incidents. Longitudinal data suggest that repeated exposure may have cumulative effects.

### 4.6 Summary of findings

See Table 3.

**Table 3:** Summary of neuroimaging findings across included studies.

Aspect	Summary
Cognitive Outcomes	Children exposed to severe hypoglycemia (SH) commonly experience deficits in memory, attention, and processing speed.
Structural Brain Changes	There is a reduction in gray matter in the hippocampus and prefrontal cortex, along with disruptions in white matter.
Functional Brain Changes	Brain activation patterns are altered during tasks related to executive function and memory.
Age Effects	Exposure to SH in early childhood (before age 6) is associated with more enduring deficits.
Frequency Effects	Repeated episodes of SH lead to increased severity of cognitive impairments.
Confounding Variables	Factors such as glycemic variability, duration of the disease, and socioeconomic status influence the outcomes.

## 5 Discussion

### 5.1 Overview of main findings

This systematic review thoroughly examined twenty peer-reviewed studies on the neurodevelopmental effects of severe hypoglycemia (SH) in children with type 1 diabetes mellitus (T1DM). The evidence indicates that severe and recurrent episodes of SH are linked to significant cognitive and structural brain changes, especially when they occur during early childhood (under six years old) (13).

The most frequently reported areas of impairment include memory, attention, and processing speed, with several studies noting a decrease in intelligence quotient (IQ) by 4–6 points compared to diabetic controls who were not exposed to SH (14). Neuroimaging studies supported these findings, showing reduced gray matter volumes in the hippocampus and structural abnormalities in white matter pathways critical for learning and executive functions.

However, not all studies observed significant effects. Some indicated only minor or reversible impairments, suggesting that the neurodevelopmental impact of SH may vary based on the frequency, timing, duration, and severity of episodes, as well as individual factors like genetic predisposition, metabolic control, and social support.

### 5.2 Comparison with previous literature

The findings of this review are consistent with and expand upon previous research from both clinical and experimental studies. Animal research has long shown that prolonged neuroglycopenia can lead to neuronal death and oxidative stress, particularly affecting the hippocampus and cerebral cortex—areas that are highly sensitive to glucose shortages. When applied to human populations, neuroimaging results in children with T1DM reveal similar vulnerabilities.

Earlier reviews [e.g., (2, 12)] also identified mild but consistent cognitive declines in T1DM, often linked to SH. This review builds on those earlier findings by incorporating newer imaging studies (2015–2024) and advanced cognitive evaluations, demonstrating that even minor metabolic disruptions during early neurodevelopment can lead to significant and lasting effects.

Interestingly, while numerous studies have confirmed changes in neurocognition, some—like Gonder-Frederick et al. (11)—found no overall reduction in IQ. These differences may be due to variations in study methodologies, small sample sizes, or the brain's ability to adapt through neural plasticity. Additionally, advancements in diabetes management technologies, such as continuous glucose monitoring and insulin pumps, may reduce the risks associated with severe hypoglycemia (SH) compared to earlier groups.

### 5.3 The impact of age at onset

The age at which hypoglycemia occurs has consistently been identified as a significant factor influencing neurodevelopmental outcomes (15). Children who experienced severe hypoglycemia before the age of six showed the most significant cognitive deficits, particularly in verbal memory and attention. This observation supports the “critical window hypothesis,” which suggests that the brain is most vulnerable metabolically during early developmental stages characterized by rapid synaptic growth and myelination.

Neuroimaging studies support this notion: early episodes of SH have been associated with atrophy in the hippocampus and thinning of the cortex, especially in the temporal and prefrontal areas that are crucial for memory and executive functions (16). While it remains unclear whether these changes can be reversed, longitudinal studies indicate that improved glycemic control may lead to some degree of neural recovery, underscoring the potential for neuroplastic adaptation.

### 5.4 The frequency and severity of hypoglycemia

The cumulative impact of repeated episodes of SH has also been identified as a key factor affecting outcomes (17). Children who experienced multiple episodes of SH each year showed greater cognitive impairments compared to those who had isolated incidents. This dose-response relationship indicates that repeated instances of glucose deprivation may have cumulative neurotoxic effects.

Some mechanistic explanations have been proposed in literature; however, current human evidence does not confirm specific biological pathways. Therefore, interpretations remain speculative.

### 5.5 Neuroimaging correlates

It is important to note that most neuroimaging studies were cross-sectional. Therefore, they cannot determine causality, directionality, or permanence of the observed differences.

Recent advancements in neuroimaging have provided objective evidence that supports cognitive findings. Structural MRI studies have consistently shown reductions in volume in the hippocampus, prefrontal cortex, and posterior parietal cortex among children with recurrent SH (18). DTI has indicated decreased fractional anisotropy in white matter tracts, suggesting compromised integrity of connectivity between brain regions.

Functional MRI (fMRI) results further revealed altered patterns of neural activation during tasks involving memory, inhibition, and attention (19). Instead of the typical activation in fronto-parietal circuits, children exposed to SH displayed compensatory hyperactivation in other cortical areas, which may indicate inefficient neural recruitment necessary to maintain performance.

The neuroimaging results collectively indicate that severe hypoglycemia (SH) does not just cause temporary metabolic changes; it may also play a role in the microstructural and functional reorganization of the developing brain.

### 5.6 Confounding factors

Although SH seems to have significant neurodevelopmental effects, various confounding factors might also affect the observed outcomes.

These factors include:

Chronic Hyperglycemia (HbA1c Levels)
  High HbA1c is independently associated with:
  Executive dysfunction
  Slower processing speed
  Reduced white matter integrity
  Because children with frequent SH sometimes also have high glycemic variability, separating the effect of SH from chronic hyperglycemia is challenging.
Glycemic Variability: Emerging evidence suggests that glucose swings—regardless of SH—may influence cognitive outcomes (20). Few studies adjusted adequately for variability metrics.
Socioeconomic Status (SES): Up to half of the included studies lacked sufficient adjustment for SES, which influences: (Health literacy, Diabetes management, Access to technology, Cognitive development independent of diabetes)
Duration of Diabetes: Longer diabetes duration is associated with greater cumulative metabolic exposure.
Diabetic Ketoacidosis (DKA) Episodes: DKA has independent neurocognitive effects (e.g., memory, attention), yet many studies did not adjust for prior DKA history.

The majority of studies did not control for all key confounding factors simultaneously, limiting the ability to isolate the specific contribution of SH to neurodevelopmental differences.

The interaction between SH and chronic hyperglycemia may contribute jointly to neurodevelopmental outcomes, and few studies were designed to disentangle these effects.

### 5.7 Comparison between pre-technology and modern technology

Older cohorts (2000–2010):

Higher SH rates (2–7 episodes/year)
Less automated insulin adjustment
Less parental real-time monitoring
More pronounced cognitive associations

Modern cohorts (2015–2025):

SH rates <1 episode/year
Wider CGM and hybrid closed-loop pump use
Improved rapid-acting insulin profiles
Smaller or absent cognitive differences

This comparison highlights the positive impact of diabetes technology on neurodevelopmental risk reduction.

### 5.8 Clinical implications

The findings carry significant clinical and public health implications. They emphasize the need for preventive measures that reduce the frequency and severity of SH without compromising glycemic control. These measures include:

- Implementing real-time continuous glucose monitoring (CGM) with predictive alerts.
- Utilizing hybrid closed-loop insulin pump systems that automatically adjust insulin delivery to prevent hypoglycemia.
- Educating parents and caregivers on the early recognition and management of hypoglycemia symptoms.
- Establishing age-appropriate glycemic targets, especially for young children who may not be able to report early warning signs.

Additionally, routine neurocognitive screening is advised for children with Type 1 Diabetes Mellitus (T1DM) who experience recurrent SH, enabling early identification of subtle deficits. Early interventions, such as cognitive training, educational support, and psychological counseling, may help mitigate long-term effects and enhance academic performance.

### 5.9 Implications for future research

Despite significant advancements, there are still considerable gaps in understanding the specific mechanisms and long-term outcomes of SH-related neurodevelopmental effects. Future research should concentrate on:

Conducting longitudinal, multi-center cohort studies that track children from diagnosis through adolescence to adulthood to assess whether the early effects of SH persist or diminish over time.
Integrating multimodal neuroimaging techniques, including MRI, DTI, and fMRI, to explore the relationships between brain structure and function.
Employing standardized neuropsychological assessment tools across studies to enable meta-analytic comparisons.
Investigating genetic and neuroprotective factors that may provide resilience against SH-induced damage.
Investigation of interventional trials examining whether technologies like hybrid closed-loop systems can lower neurodevelopmental risks.

By addressing these issues, future studies can create stronger causal connections and provide more accurate guidelines for managing diabetes in children.

### 5.10 Review strengths and limitations

#### 5.10.1 Strengths

Comprehensive search across four databases
Inclusion of both cognitive and neuroimaging evidence
Clear PICOS structure
Adherence to PRISMA guidelines

#### 5.10.2 Limitations

This systematic review has its own limitations.

The variability among the studies included—regarding definitions of severe hypoglycemia (SH), outcome measures, and statistical methods—restricted the possibility of conducting a meta-analysis.

Some studies did not provide comprehensive data on glycemic control metrics (such as HbA1c and variability indices), which could obscure the relationships being examined.
There may be publication bias, as studies with null findings might be less represented in the existing literature.
Additionally, cognitive assessment tools differ across cultural settings, which could affect performance results.

Nonetheless, by integrating evidence from various populations and methodologies, this review offers the most thorough and current understanding of the neurodevelopmental effects of SH in children with type 1 diabetes mellitus (T1DM).

### 5.11 Discussion summary

In conclusion, evidence indicates that severe hypoglycemia in childhood—particularly when it occurs repeatedly or early in the disease—can result in significant neurocognitive and neurostructural changes. The most compelling evidence points to impairments in memory, attention, and processing speed, linked to observable changes in the hippocampus and prefrontal cortex as seen in neuroimaging studies. While some recovery or compensatory mechanisms may take place, prevention is crucial. Future longitudinal and interventional research is vital to determine the reversibility of these effects and to refine clinical guidelines for glycemic management in pediatric diabetes.

## 6 Conclusion

This systematic review has compiled and critically assessed evidence from the past twenty years regarding the neurodevelopmental consequences of severe hypoglycemia (SH) in children with type 1 diabetes mellitus (T1DM). The overall findings strongly suggest that severe or recurrent hypoglycemic episodes, especially those occurring early in childhood, are linked to negative neurocognitive and neurostructural outcomes.

Although associations were observed, the predominantly observational nature of included studies prevents causal conclusions.

## 7 Key conclusions

### 7.1 Cognitive effects

Severe hypoglycemia is consistently associated with impairments in attention, working memory, executive function, and processing speed. Children who experience recurrent SH typically have lower full-scale IQ scores compared to their peers who have not been exposed.

### 7.2 Age dependency

The timing of SH is a significant factor influencing its neurodevelopmental effects. Exposure before the age of six—during critical brain development stages—poses the highest risk for long-term cognitive deficits.

### 7.3 Neurostructural connections

Recent neuroimaging research has revealed significant evidence of hippocampal shrinkage, thinning of the cortex, and compromised white matter integrity in children who have experienced recurrent severe hypoglycemia (SH). These observations align with the cognitive areas that are most impacted, indicating a direct neurobiological connection.

### 7.4 Potential for recovery and adaptability

Although some impairments may be long-lasting, others seem to be partially reversible with better management of blood sugar levels, educational assistance, and cognitive rehabilitation. The developing brain's neural plasticity may facilitate compensatory processes that help reduce lasting damage.

### 7.5 Clinical significance

Preventing severe hypoglycemia should be a primary clinical focus, especially for young children who may not recognize the signs of low blood sugar.

Implementing continuous glucose monitoring (CGM), automated insulin delivery systems, and educating families are the most effective strategies for prevention.

Regular neurocognitive evaluations are advised for children who frequently experience SH episodes to enable timely interventions.

### 7.6 Research directions

Future longitudinal studies should aim to:

Establish neuroglycopenia thresholds that can predict long-term cognitive impairment.
Investigate how severe hypoglycemia interacts with chronic high blood sugar levels in influencing neurodevelopment.
Employ multimodal imaging and standardized assessments to improve comparability across studies.

In summary, this systematic review concludes that severe hypoglycemia is not a trivial occurrence in pediatric diabetes; instead, it poses a significant risk to neurodevelopment that necessitates proactive prevention, careful monitoring, and collaborative management. Achieving optimal neurocognitive outcomes for children with Type 1 Diabetes Mellitus (T1DM) will require a careful balance between stringent blood sugar control and strategies that protect the developing brain.

## Data Availability

The raw data supporting the conclusions of this article will be made available by the authors, without undue reservation.
